# The optimal combination of cooling and equilibration durations, along with the addition of melatonin, gamma-oryzanol, and canthaxanthin, for improving swamp buffalo semen cryopreservation quality

**DOI:** 10.14202/vetworld.2024.2950-2956

**Published:** 2024-12-26

**Authors:** Wilasinee Inyawilert, Yu-Jing Liao, Oswald Nfor Ndi, Koranit Pradithera, Atchawut Saengtun, Sureeporn Saengwong, Payungsuk Intawicha, Kunlayaphat Wuthijaree, Vorawatt Hanthongkul, Kaikaew Kamdee, Anurak Khieokhajonkhet, Chalothon Amporn, Attapol Tiantong, Chompunut Lumsangkul

**Affiliations:** 1 Department of Agricultural Science, Faculty of Agriculture Natural Resources and Environment, Naresuan University, Phitsanulok, Thailand; 2 The Center for Agricultural Biotechnology, Naresuan University, Phitsanulok, Thailand; 3 Genetics and Physiology Division, Taiwan Livestock Research Institute, Ministry of Agriculture, Tainan, Taiwan; 4 Department of Public Health, Institute of Public Health, Chung Shan Medical University, Taichung, Taiwan; 5 Program of Animal Science, School of Agriculture and Natural Resources, University of Phayao, Phayao, Thailand; 6 Phitsanulok Artificial Insemination and Biotechnology Research Center, Phitsanulok, Thailand; 7 Department of Veterinary Technology, Kalasin University, Kalasin, Thailand; 8 Faculty of Animal Sciences and Agricultural Technology, Silpakorn University, Phetchaburi IT Campus, Cha-Am, Phetchaburi, Thailand; 9 Department of Animal Science, National Chung Hsing University, Taichung, Taiwan

**Keywords:** antioxidant, cryopreservation, equilibration, semen, and swamp buffalo

## Abstract

**Background and Aim:**

The success of semen cryopreservation relies on several aspects, including breed, age, season, collection method, extender composition, cooling rate, equilibration period, freezing rate, and thawing rate. This study aimed to investigate the effects of cooling and equilibration duration, as well as the addition of antioxidants to the semen extender, on the cryopreservation of swamp buffalo semen.

**Materials and Methods:**

Semen collected from swamp buffalo bulls was subjected to four different conditions: (T1) 2-h cooling and 2-h equilibration, (T2) 1.5-h cooling and 1.5-h equilibration, (T3) 1-h cooling and 1-h equilibration, and (T4) 0.5-h cooling and 0.5-h equilibration. Spermatozoa motility was evaluated using a computer-assisted semen analyzer. Moreover, this study also investigated the effect of antioxidant supplementation during cryopreservation using tris-citrate egg yolk extenders enriched with various antioxidants: Control (Con), 1 mM melatonin (ML), 0.5 mM gamma-oryzanol (GO), 10 μM canthaxanthin (CX), 1 mM melatonin + 0.5 mM gamma-oryzanol (ML + GO), and 1 mM melatonin + 10 μM canthaxanthin (ML + CX).

**Results:**

Results showed that the (T1) 2-h cooling and 2-h equilibration and (T2) 1.5-h cooling and 1.5-h equilibration groups achieved higher progressive motility than the (T3) 1-h cooling and 1-h equilibration and (T4) 0.5-h cooling and 0.5-h equilibration groups. The ML-treated group exhibited superior progressive motility and total motility.

**Conclusion:**

The optimal approach for cryopreserving swamp buffalo bull semen involves a 1.5-h cooling period followed by a 1.5-h equilibration period, with the incorporation of ML into the semen extender.

## Introduction

Artificial insemination is a critical technique for the efficient transfer of superior genetic material from a selected number of outstanding males to several females [1, 2]. This method is fundamental to genetic improvement in animals, particularly through the use of cryopreserved semen in ruminants. The success of cryopreservation is significantly influenced by the development of effective cryopreservation techniques, which are crucial for maintaining sperm viability during the freeze-thaw cycle [3–5]. Semen cryopreservation involves several steps: Cooling, equilibration, freezing, and thawing. The process typically begins by cooling the semen to a temperature between 4°C and 5°C, followed by an equilibration period that can last from 0 to 24 h at this temperature before freezing [6, 7]. Equilibration allows sperm to adapt to lower temperatures, facilitating the penetration of cryoprotectants through the cell membrane and decreasing the cellular water content. This process minimizes damage caused by ice crystal formation during the freeze-thaw process [8]. It is commonly recommended that a period of 2–4 h is optimal for cryoprotectant penetration, although the exact duration depends on the specific characteristics and efficacy of the cryoprotectant with regard to sperm quality [9, 10]. Moreover, cryopreservation significantly compromises semen quality, often by over 50%. This decline is largely attributed to several factors, including cold shock, cooling rate, ice crystal formation, diluent composition, and osmotic stress, which affect the functional condition of surviving cells [11–14].

Furthermore, the freezing process can cause considerable sperm damage, leading to apoptosis, membrane lipid peroxidation (LPO), mitochondria disruption, and DNA damage, all of which are induced by reactive oxygen species (ROS) and oxidative stress [2, 15, 16]. Previous research has examined various antioxidants that improve the quality of post-thaw. Agents such as glutathione, resveratrol, vitamin E, bovine serum albumin, methionine, carnitine, butylated hydroxytoluene, melatonin (ML), inositol, spirulina maxima extract, selenium, cysteine, taurine, gamma-oryzanol, and canthaxanthin (CX) have been identified as potential enhancers of semen quality in domestic animals [16–26]. Melatonin, also known as N-acetyl-5-methoxy tryptamine, is produced by the pineal gland. It acts as a powerful antioxidant that neutralizes harmful free radicals, even at low concentrations, and is commonly used to reduce the formation of ROS and LPO [20]. Recent studies have demonstrated the protective effects of melatonin supplementation at various concentrations on spermatozoa during the freezing and thawing process in swamp buffalo. Specifically, the addition of 0.1, 0.5, 1.0, and 2.0 mM melatonin to the semen extender improved sperm viability and motility. Notably, supplementation with 1.0 mM melatonin demonstrated the most beneficial outcomes among all measured parameters [27]. Gamma-oryzanol (GO), derived from rice bran oil, comprises ferulate esters of triterpene alcohols and phytosterols. It plays a crucial role in enhancing the powerful antioxidant effects against cholesterol oxidation and safeguarding against LPO, thus surpassing the actions of vitamin E. For instance, the addition of 0.5 mM gamma-oryzanol to a tris-citrate egg yolk extender used to treat swamp buffalo semen significantly enhanced progressive motility [28–31]. Canthaxanthin (CX), also known as β, β-carotene 4,4’-dione, belongs to the carotenoid family. It is an exceptionally effective free radical scavenger and antioxidant, surpassing other carotenoids because of its keto group, which is critical in preventing LPO in spermatozoa [20, 32, 33]. For example, supplementation with 10 and 25μM of canthaxanthin in a Tris-egg yolk extender for ram semen cryopreservation effectively preserves the viability of ovine sperm [34].

Buffalo, an indigenous species, plays a crucial role in Thailand’s livestock industry, and its population has been sustained through steady reproduction rates. Buffalo sperm are more vulnerable to oxidative stress than cattle sperm [35, 36]. Many studies have examined the use of antioxidants alongside extenders to enhance semen cryopreservation. Despite the widespread use of melatonin, gamma-oryzanol, and canthaxanthin throughout numerous domains, studies on their comparative effects and the combined impacts of melatonin with canthaxanthin or gamma- oryzanol on the preservation of buffalo sperm are limited.

Hence, we investigated the effects of cooling and equilibration periods to minimize the time required for cooling and equilibration while maintaining the quality of sperm cryopreservation. Moreover, the combined use of antioxidants may provide new insights into semen cryopreservation.

## Materials and Methods

### Ethical approval

This study was approved by the Institutional Animal Use and Care Committee (IACUC) of Naresuan University, Thailand (no. NU-AG670401).

### Study period and location

This study was conducted from March 2024 to June 2024 at the Faculty of Agriculture, Natural Resources and Environment, Naresuan University, Phitsanulok, Thailand, Big Ice Swamp Buffalo Farm, Phitsanulok, Thailand, and the Phitsanulok Artificial Insemination and Biotechnology Research Center, Phitsanulok, Thailand.

### Animals

Six adult swamp buffalo bulls aged 4–11 years were employed in this study and were maintained under optimal feeding and management conditions. We used variables associated with clinical health and libido for selection. Each bull received a daily ration of 3 kg of concentrated supplements, along with unrestricted access to dry grass or rice straw and fresh water. Furthermore, the buffaloes were allowed to freely graze in grassy fields during daylight hours.

### Semen collection

Semen was collected once a week for 2 consecutive weeks using an artificial vagina. The ejaculates from individual bulls were evaluated against minimum criteria, which required at least 70% motility and a sperm concentration of no <500 × 10^6^ sperm/mL.

### Preparation of a semen extender

The tris-citric egg yolk extender was composed of 200 mM tris-(hydroxymethyl)-aminomethane, 70 mM citric acid, 55 mM fructose, 20% (v/v) egg yolk, 7% (v/v) glycerol, benzylpenicillin at 1000 IU/mL, and streptomycin sulfate at 1000 μg/mL. The pH of the extender solution was adjusted to 6.9. The semen was then diluted to a specific concentration using the extender.

### Cooling and equilibration conditions

The sperm concentration was adjusted to 20 × 10^6^ sperm/mL. The diluted semen was placed in 0.25 mL straw and gradually cooled to 4°C. The experimental design included four groups: The first group (T1) involved a 2-h cooling at 4°C followed by a 2-h equilibration at 4°C, resulting in a total of 4 h. The second group (T2) involved 1.5 h of cooling at 4°C and 1.5 h of equilibration at 4°C, totaling 3 h. The third group (T3) involved 1 h of cooling at 4°C and 1 h of equilibration at 4°C, totaling 2 h. The fourth group (T4) involved 0.5 h of cooling at 4°C and 0.5 h of equilibration at 4°C, totaling 1 h. After the respective cooling and equilibration periods, the semen straws were placed 5 cm above liquid nitrogen (LN2) for 15 min in a styrofoam box, followed by immersion in LN2 for long-term storage at –196°C.

### Supplementation with antioxidants

To improve semen quality, antioxidants including ML (M5250; Sigma-Aldrich), CX (11775; Sigma-Aldrich), and GO (Tokyo Chemical Industry Co., Ltd. lot.5ZZYLPJ) were supplemented in the extender. ML and CX were prepared in dimethyl sulfoxide (DMSO), whereas GO was prepared in King rice bran oil (King Rice Oil Group, Thailand). The supplements were added to the extenders at the following final concentrations: 10 μM CX, 0.5 mM GO, and 1 mM ML. Melatonin and canthaxanthin were solubilized in DMSO. The final concentration of DMSO applied to the extender was 0.1%. Gamma-oryzanol is soluble in oil. The sperm were divided into six groups: Control (Con), CX, GO, ML, ML+CX, and ML+GO. The semen concentration in each group was adjusted to 20 × 10^6^ sperm/mL. The diluted semen was placed in 0.25 mL straw, gradually cooled to 4°C over a period of 1.5 h, and then equilibrated at this temperature for an additional 1.5 h. The semen straws were then placed 5 cm above LN2 for 15 min in a styrofoam box and then promptly immersed in LN2 for long-term storage at –196°C.

### Assessment of sperm movement characteristics

Three semen straws were thawed in a water bath at 37°C for 30 s. Thawed semen samples were assessed for sperm motility, velocity distribution, and kinematics using computer-assisted sperm analysis with the IVOS II system (Hamilton Thorne, Beverly, MA, USA). For analysis, 6 μL of thawed semen from each group was applied to pre-heated slides (Leja Slides, SCA^®^, Spain) maintained at 37°C. The following parameters were measured: Total motility (Tot motile), static sperm (Static), progressive motility (P motile), slow motility (Slow), straight-line velocity (VSL), curve-line velocity (VCL), average path velocity (VAP), distance average path, distance straight line, distance curve line, linearity (LIN), straightness (STR), beat cross frequency (BCF), wobble (WOB), and amplitude of lateral head displacement (ALH).

### Statistical analysis

Data were analyzed using analysis of variance (ANOVA). The General Linear Model technique from SAS (SAS Enterprise Guide 4.1, SAS Institute Inc., and Cary, NC, USA) was used to perform the ANOVA. The significance of the variations was assessed using Duncan’s multiple-range test. A p < 0.05 was considered statistically significant.

## Results

### Effects of cooling and equilibration duration on the quality of frozen-thawed semen in swamp buffalo

Sperm parameters were evaluated following thawing, cooling, and equilibration. The effects of different cooling and equilibration durations on the post-thaw sperm quality of swamp buffalo are presented in Table-1. The results indicated that cooling and equilibration periods of 4 h (T1) (18.51 ± 0.81) and 3 h (T2) (17.90 ± 1.69) resulted in significantly higher progressive motility compared with the 2-h (T3) (12.59 ± 1.23) and 1-h (T4) (6.92 ± 0.71) (p < 0.05) durations. Moreover, the 4-h (T1) (39.56 ± 1.73) and 3-h (T2) (34.34 ± 2.42) periods exhibited significantly higher total motility than the 2-h (T3) (27.45 ± 2.71) and 1-h (T4) (16.00 ± 1.54) periods (p < 0.05). Interestingly, T1 demonstrated significantly lower values for VCL and ALH compared to T2, T3, and T4 (p < 0.05). The parameters STR, LIN, and WOB were significantly higher in T1 than in T2, T3, and T4 (p < 0.05). Nevertheless, no significant differences were observed in VAP and VSL among all groups.

**Table-1:** Effects of cooling and equilibration time on the cryopreservation of swamp buffalo semen.

Parameters	Cooling and equilibration time			

	T1 (4 h)	T2 (3 h)	T3 (2 h)	T4 (1 h)
Progressive motility (%)	18.51 ± 0.81^a^	17.90 ± 1.69^a^	12.59 ± 1.23^b^	6.92 ± 0.71^c^
Total motility (%)	39.56 ± 1.73^a^	34.34 ± 2.42^a^	27.45 ± 2.71^b^	16.00 ± 1.54^c^
Static motility (%)	60.44 ± 1.73^c^	65.66 ± 2.42^c^	72.55 ± 2.71^b^	84.00 ± 1.54^a^
VAP [μm/s]	67.03 ± 1.26	74.53 ± 2.41	70.81 ± 3.01	72.33 ± 2.83
VSL [μm/s]	54.57 ± 1.25	54.20 ± 1.43	51.47 ± 1.87	50.55 ± 1.79
VCL [μm/s]	117.44 ± 2.35^b^	139.81 ± 5.35^a^	134.47 ± 6.40^a^	139.93 ± 5.59^a^
ALH [μm]	5.40 ± 0.15^b^	7.01 ± 0.22^a^	6.74 ± 0.26^a^	6.89 ± 0.24^a^
BCF [Hz]	31.15 ± 0.62^a^	29.38 ± 0.24^b^	29.38 ± 0.40^b^	29.98 ± 0.49^ab^
STR	80.50 ± 0.82^a^	72.93 ± 1.24^b^	74.21 ± 1.25^b^	72.13 ± 1.19^b^
LIN	49.13 ± 0.99^a^	42.09 ± 0.88^b^	43.71 ± 1.32^b^	40.99 ± 0.86^b^
WOB	58.93 ± 0.66^a^	55.58 ± 0.40^bc^	56.50 ± 0.89^b^	54.50 ± 0.42^c^

^a,b^Different superscripts within the same row indicate significant differences at p *<* 0.05. BCF=Beat cross frequency, STR=Straightness, WOB=Wobble, ALH=Amplitude of lateral head displacement, VSL=Straight-line velocity, LIN=Linearity

### Effects of antioxidants on semen cryopreservation

Since the T2 group with 1.5-h cooling and 1.5-h equilibration showed the optimal conditions and time frame for semen cryopreservation, this setup was used in subsequent experiments. To improve the quality of thawed sperm in swamp buffaloes, various supplements were added to the extender, including CX, GO, ML, ML+CX, and ML+GO. The results indicated that ML significantly enhanced progressive motility (24.00 ± 2.44) compared with the control (16.16 ± 1.72), CX (14.15 ± 1.70), GO (17.37 ± 2.57), ML+CX (15.95 ± 2.44), and ML+GO groups (13.46 ± 1.35) (p < 0.05) (Table-2). Total motility in the ML group (44.47±2.27) did not differ from that in the GO group (40.13 ± 3.62) but was significantly higher than that in the control (30.58 ± 1.97), CX (32.10 ± 1.34), ML+CX (36.43 ± 1.94), and ML+GO groups (31.49 ± 3.75) (p < 0.05). The VCL values were significantly higher in the ML group (131.63 ± 6.64) than in the GO (104.68 ± 6.79) and ML+CX groups (97.23 ± 9.50) (p < 0.05). However, there were no significant differences between the ML group (131.63 ± 6.64) and control (122.05 ± 4.98), CX (111.29 ± 7.93), or ML+GO groups (113.72 ± 4.16). The VAP, VSL, and BCF did not exhibit significant differences between the ML and other groups (Table-2).

**Table-2:** Comparison of the efficiency of CX, GO, ML, ML + CX, and ML + GO in the cryopreservation of swamp buffalo semen.

Parameters	Con	CX	GO	ML	ML + CX	ML + GO
Progressive motility (%)	16.16 ± 1.72^b^	14.15 ± 1.70^b^	17.37 ± 2.57^b^	24.00 ± 2.44^a^	15.95 ± 2.44^b^	13.46 ± 1.35^b^
Total motility (%)	30.58 ± 1.97^c^	32.10 ± 1.34^c^	40.13 ± 3.62^ab^	44.47 ± 2.27^a^	36.43 ± 1.94^bc^	31.49 ± 3.75^c^
Static motility (%)	69.42 ± 1.97^a^	67.90 ± 1.34^a^	59.88 ± 3.62^c^	55.53 ± 2.27^c^	63.57 ± 1.94^ab^	68.51 ± 3.75^a^
VAP [μm/s]	66.49 ± 2.53	60.10 ± 3.84	59.91 ± 3.53	70.55 ± 3.59	59.59 ± 4.81	62.54 ± 1.94
VSL [μm/s]	51.00 ± 2.32	45.26 ± 2.98	47.03 ± 3.47	54.18 ± 3.03	48.11 ± 3.98	46.14 ± 1.42
VCL [μm/s]	122.05 ± 4.98^ab^	111.29 ± 7.93^abc^	104.68 ± 6.79^bc^	131.63 ± 6.64^a^	97.23 ± 9.50^c^	113.72 ± 4.16^abc^
ALH [μm]	6.31 ± 0.14^ab^	5.90 ± 0.33^bc^	5.85 ± 0.29^ab^	6.74 ± 0.25^a^	5.27 ± 0.38^c^	6.38 ± 0.14^ab^
BCF [Hz]	29.41 ± 0.53	28.08 ± 0.53	27.93 ± 0.48	28.79 ± 0.57	29.12 ± 0.74	29.81 ± 0.49
STR	75.50 ± 1.19^bc^	77.66 ± 1.75^ab^	79.22 ± 1.68^ab^	77.14 ± 0.94^b^	81.77 ± 1.72^a^	72.91 ± 0.57^c^
LIN	45.82 ± 1.32^bc^	47.54 ± 2.46^bc^	50.86 ± 2.54^ab^	45.84 ± 1.29^bc^	55.75 ± 3.78^a^	43.20 ± 0.69^c^
WOB	57.83 ± 1.01^b^	59.27 ± 1.78^b^	61.70 ± 1.87^ab^	57.43 ± 0.96^b^	66.08 ± 3.03^a^	57.18 ± 0.54^b^

^a,b^Different superscripts within the same row indicate significant differences at p *<* 0.05. BCF=Beat cross frequency, STR=Straightness, WOB=Wobble, ALH=Amplitude of lateral head displacement, VSL=Straight-line velocity, LIN=Linearity

## Discussion

Sperm cryopreservation is a complex process involving osmotic, thermal, and mechanical stressors that impact spermatozoa. Stressors during dilution, cooling, equilibration, freezing, and thawing induce harmful changes in sperm function. Such changes are associated with osmotic stress, exposure to freezing temperatures, ice crystal formation within cells, excessive ROS production, and disruption of antioxidant mechanisms [11, 19, 37–39]. Hence, optimizing the cooling and equilibration processes is crucial to ensure successful sperm freezing. An appropriate cooling rate is essential for developing an optimal cryopreservation technology. We hypothesized that the equilibration duration might be influenced by the initial cooling time, which might affect the long-term survival of post-thaw sperm. The equilibration period directly affects sperm stabilization in a prolonged environment, ensuring the maintenance of homeostasis, osmotic balance, and cryo-tolerance. Proper cooling rates and equilibration times enable sperm to withstand physical, osmotic, and temperature stress during freezing [6, 40, 41].

Various cooling rates, including rapid, moderate, and slow cooling processes, have been investigated across species, along with the corresponding equilibration periods. Ahmad *et al*. [6] investigated the impact of two cooling rates: moderate cooling, where the temperature was reduced from 37°C to 4°C over 90 min, and rapid cooling, where the temperature was lowered to 4°C within 15 min. The results suggested that moderate-intensity cooling produced better sperm parameters than rapid cooling. Further studies combining cooling rates (moderate and rapid) with equilibration times (0, 2, 4, and 8 h), revealed that moderate cooling followed by 2–8 h of equilibration improves the quality of post-thaw buck sperm [6]. The relationship between cooling and equilibration time is crucial for establishing a strategy that determines the sequence and timing of cryopreservation. Our study examined the influence of cooling and equilibration duration on the post-thaw quality of swamp buffalo sperm. Our findings demonstrate that cooling and equilibration periods of 4 h (T1) and 3 h (T2) enhanced progressive motility compared with periods of 2 h (T3) and 1 h (T4). A marked difference in total motility was noted between cooling and equilibration periods of 4 h (T1) and 3 h (T2) versus 2 h (T3) and 1 h (T4). Interestingly, T1 showed significantly lower values for VCL and ALH parameters compared to T2, T3, and T4 (p < 0.05). The STR, LIN, and WOB parameters were significantly higher in T1 than in T2, T3, and T4 (p < 0.05). However, no significant differences were observed in the VAP and VSL parameters in T1 among the groups.

The cooling mechanism reduces the metabolic activity of sperm cells. Inadequate cooling can disrupt membrane integrity through protein disarrangement, ion channel interference, ROS generation, and the reduction of mitochondrial membrane potential [12, 40, 42]. Seminal plasma usually contains both enzymatic and non-enzymatic antioxidants. Nevertheless, the defense against oxidative stress is significantly diminished when semen is diluted in extenders before storage. In addition, high levels of polyunsaturated fatty acids make the sperm cell’s outer membrane very vulnerable to oxidative stress, particularly LPO caused by ROS, due to an imbalance between ROS levels and the natural antioxidant activity of the sperm [43–45]. ROS adversely affects spermatozoa, leading to decreased sperm viability, motility, and fertilization [46, 47]. Therefore, the use of exogenous antioxidants to regulate ROS balance and protect spermatozoa from oxidative damage during preservation [16, 27, 28, 48]. However, the efficacy of antioxidants such as resveratrol, iodixanol, cysteamine, glutathione, quercetin, methionine sulfoxide reductase A, l-arginine, catalase, melatonin, gamma-oryzanol, and canthaxanthin in maintaining sperm quality during semen preservation remains debated [20, 22, 27, 28, 49–52]. Our previous studies discovered that supplementation with 1 mM ML and 0.5 mM GO enhances the quality of post-thaw swamp buffalo semen [27, 28]. A recent study indicated that supplementation with 10 and 25 μM of CX in a Tris-egg yolk extender for ram semen cryopreservation was protective for ovine sperm [34]. In this study, we aimed to enhance the quality of thawed swamp buffalo sperm by adding various antioxidant supplements to the extender, focusing on ML, CX, and GO. The results indicated that ML supplementation significantly increased progressive and total motility (p < 0.05) (Table-2). A previous study by Bhalothia *et al*. [20] on supplementing ML and CX in a Tris-based extender indicated that these antioxidants enhance the storage life of ram semen at 4°C. Our findings indicate that ML supplementation alone significantly improves the cryopreserve ability of swamp buffalo semen. Nevertheless, we note that the co-supplementation of ML with CX and GO may have a negative effect on the quality of cryopreserved swamp buffalo spermatozoa.

## Conclusion

The optimal method for preserving spermatozoa in the cryopreservation of swamp buffalo semen involves a 3-h cooling period followed by a 4-h equilibration period. The addition of ML to the semen extender for cryopreservation greatly improves the movement of sperm after thawing. Additional investigation for ROS and pregnancy rates is recommended to validate these findings.

## Authors’ Contributions

WI: Designed the study. WI, KP, AS, VH, and KK: Performed the study. WI, KW, SS, YJL, ONN, and AT: Analyzed the data. SS, PI, AK, CL, and CA: Facilitated the research and provided guidance on the study. WI, YJL, ONN, AT, SS, PI, AK, CL, and CA: Revised and edited the manuscript. All authors have read and approved the final manuscript. All authors have read and approved the final manuscript.
